# Copper and Cholera

**Published:** 1885-11

**Authors:** 


### Copper and Cholera.

M. Burq has renewed his advocacy of copper by writing a note which was presented to to the French *Academe des Sciences* at a recent meeting by M. Bouley ("Gaz. hebd. de med. et de chir."). The note contains the following propositions :

1. People imbued with copper by working with the metal daily have always been exempt from cholera, with very rare exceptions.

2. Numerous experiments in certain hospital services have demonstrated that the free use of copper is sovereign against the cramps other nervous phenomena peculiar to cholera.

3. Dr. Lisle's cases (twenty-five cures in thirty-two cases), Dr. Pellarin's, Dr. Arnal's, Dr. Blondet's, Dr. Berger's, and those of others, as well as M. Burq's own experiments made at the Hotel-Dieu in 1866, in conjunction with M. Horteloup, showed that the salts of copper, administered freely by the mouth and by the rectum, and by the endermic method in the gravest cases, were the remedy *par excellence* for cholera. Of sixty-six known cases of confirmed cholera, eighteen of which were treated at the Hotel-Dieu, in which the absorption of the remedy was still possible, there were fifty-five recoveries.

[At a meeting of the *Societe de biologie* (*Ibid.*), M. Bochefontaine submitted a letter from Dr. Muston, of Montheliard, contradicting certain of M. Burq's assertions relative to cupric immunity from cholera, and stating that the workmen from Beaucourt, composing nearly the whole of the population, all work in copper, brass, iron and steel, and that they were decimated by the epidemic of 1854, although they were the very workmen that M. Burq has declared were preserved from the disease.]

---

Borax as a Preventive of Cholera.—At the same meeting (*Ibid.*) M. de Cyon called attention anew to the antiseptic properties of borax, and to the fact that it was so harmless that it could be taken into the system in quantities as great as fifteen grammes [nearly half an ounce] daily without giving rise to the least trouble, as he had announced so long ago as 1878 Since that time his confidence has but increased in the excellent qualities of the drug in all parasitic or microbic affections, and notably as a powerful preservative against cholera. Its efficiency, he says, is shown by the fact that during past epidemics of cholera the workmen employed in manufactories of boric acid have always been spared, although the neighboring population were killed in the proportion of one-third, as at Lordevello, in Italy, for instance, in 1864–'65. Taken in doses of five or six grammes daily, borax not only has a direct action on the microbes contained in the intestinal canal, but, passing into the blood, it is capable of reaching the bacilli which have gained access to that fluid. In cholera times the constipating action of the biborate of sodium is an additional argument in its favor. The author advises washing the surface of the body and the external mucous membranes with a solution of boric acid or of borax, and mixing the latter with the food and drink to the extent of ten grammes in twenty-four hours.

